# Fragile Egos and Broken Hearts: Narcissistic and Borderline Personality Traits Predict Reactions to Potential Infidelity

**DOI:** 10.3390/ijerph21101272

**Published:** 2024-09-25

**Authors:** Avi Besser, Virgil Zeigler-Hill

**Affiliations:** 1 The Interdisciplinary School for Sciences, Health and Society, Department of Communication Disorders, Hadassah Academic College, 37 Hanevi’im, St. Jerusalem 9101001, Israel; 2 Department of Psychology, Oakland University, 212A Pryale Hall, Rochester, MI 48309, USA

**Keywords:** narcissism, borderline personality, infidelity threat, negative emotional responses, romantic relationships

## Abstract

We examined the connections that narcissistic and borderline personality traits had with hypothetical responses to romantic infidelity in a sample of Israeli community members (N = 997). We distinguished between four forms of narcissism: extraverted narcissism (characterized by assertive self-enhancement), antagonistic narcissism (characterized by defensiveness and hostility), neurotic narcissism (characterized by emotional distress), and communal narcissism (characterized by attempts to emphasize superiority over others by exaggerating communal characteristics such as being extraordinarily helpful). We also measured levels of borderline personality traits. Results showed that neurotic narcissism was strongly associated with heightened negative emotional responses, particularly in high-threat infidelity scenarios, aligning with predictions regarding emotional volatility. Antagonistic and communal narcissism showed detrimental effects on relationship evaluations primarily under low-threat conditions, indicating distinct patterns of defensiveness and vulnerability. Extraverted narcissism showed no significant association with emotional responses. Borderline traits were linked to intense emotional reactions across conditions, emphasizing their broad impact on perceived relational threats. These findings suggest that while some personality traits exacerbate reactions in less severe conditions, infidelity trauma can overwhelm these differences, underscoring the potential need for personalized therapeutic approaches. Discussion is focused on the implications for understanding personality traits in relational contexts and future research directions exploring varied threat manipulations.

## 1. Introduction

Infidelity, defined as secretive sexual and/or emotional involvement outside of a committed monogamous relationship, is a prevalent issue (e.g., [1]). Estimates suggest that 20–25% of marriages experience infidelity over their lifetime [2]. Furthermore, some studies report that up to 70% of romantic relationships involve some act of infidelity at some point [3]. Infidelity can have a wide range of impacts on individuals, couples, and families, and it is recognized as one of the leading reasons for divorce [2]. Infidelity can also negatively affect the mental and physical health of the partner who remains faithful [4]. Given that infidelity presents a considerable threat to romantic relationships, it is important to understand how individual differences in personality traits may influence reactions to such betrayal.

Narcissism is a complex and multifaceted personality trait defined by traits such as self-preoccupation, grandiosity, entitlement, constant craving for admiration, and lack of empathy, and individuals with narcissistic traits often seek validation and approval through various means [5]. The link between narcissism and attitudes toward infidelity in romantic relationships is well-researched and understood—both from individual-level studies with mostly student samples (e.g., [6,7,8]) and dyadic research in long-term committed relationships (e.g., [9]). According to previous studies, individuals with narcissistic traits report more positive attitudes toward infidelity and are more prone to engage in infidelity in their romantic relationships.

Rather than being a monolithic construct, narcissism has multiple dimensions [10]. For example, the prototypical form of narcissism is *grandiose narcissism* which involves self-assurance and dominance. Those with grandiose narcissistic tendencies may feel entitled to engage in sexual activities outside their committed relationships and might use their partnerships to fulfill their self-regulatory needs (e.g., [9]). Their potential lack of empathy toward their partner’s feelings can also contribute to infidelity. In contrast to grandiose narcissism, *vulnerable narcissism* refers to a desire for affirmation and approval combined with the tendency to feel easily threatened or rejected. Individuals with vulnerable narcissistic tendencies might resort to infidelity to seek reassurance, affirmation, and attention from their romantic partners (e.g., [9]).

Overall, studies show that narcissism is positively associated with the likelihood of having affairs and the number of partners cheated on [6,11,12]. However, much less is known about the negative emotional reactions and romantic relationship investment of individuals with narcissistic personality traits when faced with the possibility of being betrayed by their romantic partner. That is, previous research has focused primarily on the infidelity committed by those with narcissistic traits rather than examining how these individuals respond when their partners are unfaithful to them.

Various personality traits, including self-criticism, dependency, grandiose and vulnerable narcissism, pathological narcissism, low self-esteem, and attachment-related anxiety and avoidance, have been shown to influence reactions to infidelity [13,14,15,16,17]. The present study focuses on the roles that narcissistic personality traits—as well as borderline personality traits—play in reactions to infidelity.

A notable study conducted by Besser and Priel (2010) [14] involved 448 Israeli community members who were randomly assigned to hypothetical scenarios intended to evoke high- or low-levels of threat in situations involving either achievement failure (i.e., being passed over for a promotion at work) or interpersonal rejection (i.e., discovering that one’s romantic partner was having a sexual affair). Participants were then asked to anticipate their reactions to their assigned hypothetical situation. The study revealed that, in the high achievement-threat group, but not in the high interpersonal-threat group, grandiose narcissism predicted more negative outcomes. In contrast, in the face of a high-level interpersonal threat, but not a high-level achievement-threat, high levels of vulnerable narcissism were associated with more negative outcomes. These findings illustrate how different types of threatening situations vary in their relevance to grandiose narcissism as compared to vulnerable narcissism. According to these findings, grandiose narcissism is associated with heightened reactivity to achievement setbacks, whereas vulnerable narcissism involves sensitivity to shaming interpersonal experiences [14]. This aligns with previous research by Besser and Priel (2009) [13] which found that higher scores on attachment anxiety, but not on attachment avoidance, were associated with stronger reactions to a vignette depicting a scenario of romantic partner’s imaginary infidelity (rejection). Moreover, higher scores on covert narcissism (vulnerable narcissism) were associated with stronger emotional responses to the induced imaginary disloyalty. Their findings indicated that covert narcissism seemed to constitute a specific aspect of attachment anxiety.

Although distinguishing between grandiose narcissism and vulnerable narcissism has been beneficial, it has been recognized that they share a common element of antagonism which has led to some confusion in the literature [18,19]. This recognition led to the development of the Trifurcated Model of Narcissism [20]. which identifies three core narcissistic traits: *extraverted narcissism*, which involves exhibitionism and self-assurance; *antagonistic narcissism*, characterized by defensiveness and aggression; and *neurotic narcissism*, associated with distress and a strong need for approval. This nuanced understanding of narcissism has prompted researchers to explore the contradictory patterns that have sometimes been observed in the literature. For example, extraverted narcissism is often characterized by certain trends (e.g., high levels of satisfaction with romantic partners), whereas antagonistic narcissism tends to exhibit different patterns (e.g., low levels of relationship satisfaction and commitment; [21]). These insights underscore the necessity of distinguishing between these aspects of narcissism to fully understand how individuals with narcissistic personality traits will respond to potential betrayal.

Communal narcissism, which is characterized by seeking admiration for perceived altruism and selflessness, has also gained notable attention in recent years [22,23]. Although communal narcissism is not addressed in the Trifurcated Model of Narcissism, incorporating communal narcissism along with extraverted, antagonistic, and neurotic forms of narcissism may be beneficial for a holistic understanding of narcissistic traits and their connections with responses to infidelity. Despite the considerable focus on extraverted, antagonistic, and neurotic narcissism due to their disruptive behaviors, communal narcissism has emerged as a distinct and relatively underexplored facet in terms of romantic relationship functioning. Exploring communal narcissism alongside the traditional dimensions enhances our grasp of narcissism’s multifaceted nature and the diverse behavioral and emotional outcomes it can produce. Furthermore, the emphasis of communal narcissism on seemingly prosocial traits, such as generosity and kindness, offers valuable perspectives on how narcissistic individuals might perceive issues like infidelity. Specifically, it provides insights into how they might view such scenarios as opportunities to exhibit the communal virtues they want to be viewed as possessing.

In addition to narcissistic personality traits, we also considered the connections that borderline personality traits have with reactions to hypothetical infidelity by one’s romantic partner. Borderline Personality Disorder (BPD) is a mental health condition marked by profound instability and variability across several life domains [24]. These include self-image, emotional reactions, interpersonal relations, and cognitive processes [25]. Conversely, borderline personality traits share characteristics associated with BPD but presents them at a lesser intensity [26]. Individuals with borderline personality traits typically experience less functional impairment compared to those who fulfill the criteria for BPD. The interpersonal difficulties that characterize those with BPD have been shown to extend to their romantic relationships (e.g., [27]). In fact, the pattern of intense and unstable romantic relationships may be one of the most useful criteria for diagnosing BPD [28].

We believed that assessing borderline personality traits along with narcissistic personality traits would offer insight into the similarities and differences in how individuals with elevated levels of these traits respond to the threat of infidelity. Borderline personality traits, which involve intense emotional volatility, fear of abandonment, and unstable relationships, may heighten the emotional distress and impulsive reactions to perceived infidelity. In contrast, narcissistic personality traits, characterized by entitlement, a need for admiration, and hypersensitivity to ego threats, may lead to reactions that focus more on personal injury and preserving self-image. Clarifying where these traits overlap and where they differ may be beneficial for predicting, assessing, and addressing the psychological impact of infidelity in individuals with these personality features.

Like narcissism, the connections between borderline personality traits and a propensity toward infidelity are well-documented (e.g., [29]). According to the *DSM-5*, various forms of impulsivity [30], including sexual impulsivity, are linked to BPD [24]. Individuals exhibiting traits of borderline personality disorder often engage in a greater number of shorter romantic relationships and display elevated levels of sexual impulsivity [31]. Those with pronounced borderline traits may experience early sexual encounters, participate in casual sexual relationships, have multiple sexual partners, and engage in high-risk sexual behaviors [32]. These characteristics may heighten their likelihood of infidelity.

Early relational difficulties with primary caregivers can hinder the development of effective emotion regulation skills, leaving those with borderline personality traits ill-equipped to manage negative emotions [33]. Consequently, they may rely on romantic partners to provide the emotional support they lack. Difficulties in regulating emotions often lead to serious challenges in interpersonal relationships [34], fostering anxious thoughts regarding the stability of their relationships and prompting behaviors such as seeking additional partners as a form of emotional security. Individuals with borderline personality traits may resort to various coping mechanisms to manage their symptoms [35] and might engage in self-sabotaging behaviors [36]. Their struggles with mentalization often lead them to express their fears through actions rather than words, resulting in behaviors that can damage their relationships [37].

However, there is limited understanding of how individuals with borderline traits respond to the perceived threat of infidelity from a romantic partner. People with these traits frequently struggle with persistent feelings of emptiness, driving them to seek relationships to alleviate these feelings [38]. They often exert significant effort to avoid feelings of real or imagined abandonment and have frantic attempts to avoid potential rejection or abandonment and display a high degree of dependence on others. Since their self-esteem is closely tied to external validation, they struggle to cope with experiences of rejection, loneliness, and separation [35]. Close interpersonal relationships are particularly challenging for those with high levels of borderline personality traits. The difficulties they face in regulating negative emotions can create significant issues for both themselves, and those around them. BPD is also associated with a greater susceptibility to maladaptive impulsive behaviors, especially in settings marked by perceived rejection or stress, and known to be associated with mate retention behaviors due to suspicious jealousy [39].

In the present study, we investigated borderline personality traits in the context of the Five Factor Model of personality which is an approach to comprehending the dimensional nature of personality disorders through a small set of basic personality traits. Research has demonstrated that each personality disorder can be understood as problematic combinations of these fundamental traits. Specifically, this perspective has been applied to BPD, which is predominantly characterized by high levels of neuroticism, with additional influences from (low) agreeableness, (low) conscientiousness, and (high) openness to experience. This conceptualization has led to the development of specialized assessment tools, such as the Five Factor Borderline Inventory (FFBI), its shortened version, the FFBI-short form (FFBI-SF), and a super short form (FFBI-SSF). These instruments are designed to evaluate subclinical levels of borderline personality traits, providing a nuanced understanding of BPD within the framework of the Five Factor Model.

Given this context, the objective of the current study is, in addition to the examination of the role of narcissistic personality traits, to investigate the associations that borderline personality traits have with emotional responses and relationship evaluations when confronted with the threat of potential betrayal by a romantic partner. To our knowledge, this study is the first to examine how individuals with borderline personality traits react to the prospect of romantic infidelity.

### Overview and Predictions

Our goal for the present study was to examine the role that narcissistic and borderline personality traits played in how individuals responded to hypothetical situations involving romantic infidelity by one’s partner. More specifically, we were interested in how individuals high in narcissistic and borderline personality traits believed they would respond if they learned that their romantic partner had been unfaithful. Investigating the responses of individuals with narcissistic and borderline traits to infidelity is crucial for a comprehensive understanding of interpersonal dynamics and psychological resilience within romantic relationships. While existing research has examined the propensity of individuals with these traits to engage in infidelity themselves [6,11,12,29], the connections that these traits have with the consequences of being cheated on by a partner have received little attention. Examining these responses can reveal how narcissistic and borderline traits influence the experience of betrayal, which could have significant implications for therapeutic processes and relationship management. Understanding these reactions is vital for developing targeted interventions that address the unique needs of individuals with elevated levels of these personality traits, potentially enhancing relationship stability and well-being.

Our predictions for antagonistic narcissism, neurotic narcissism, and borderline personality traits were similar such that we expected individuals with elevated levels of these traits to anticipate experiencing more negative emotional responses and hold more negative views of their relationships following infidelity by their partner. In essence, we expected individuals with these personality traits to be particularly prone to experiencing intense negative emotions, such as anger, when contemplating a hypothetical situation in which their romantic partner was unfaithful. Antagonistic narcissism and neurotic narcissism are characterized by heightened sensitivity to potential threats. Individuals with elevated levels of these traits may react strongly to perceived betrayal, as it may directly challenge their sense of entitlement and already uncertain feelings of self-worth. Similarly, individuals with borderline personality traits, who often struggle with fear of abandonment and emotional instability, may be likely to experience heightened distress and anger when faced with potential infidelity, which threatens their already fragile interpersonal bonds. These intense negative emotions can contribute to dissatisfaction in romantic relationships, as individuals with these personality features may become consumed by feelings of betrayal, inadequacy, and mistrust, even if actual infidelity has not occurred, further destabilizing their relationships. In contrast, we did not have clear predictions for the associations that extraverted narcissism or communal narcissism would have with anticipated responses to infidelity, but we examined these associations for exploratory purposes.

## 2. Materials and Methods

### 2.1. Participants and Procedure

The study included 1055 members of the Israeli community who volunteered to participate by responding to requests distributed through flyers in public areas and postings on various social media platforms. All questionnaires used in the present study were administered in Hebrew after being translated from the original English versions using the back-translation method. We excluded data for 58 participants due to reasons such as them failing two or more attention checks or having invariant response patterns (i.e., selecting the same response for a large percentage of the items). Out of the initial participants, the final sample consisted of 997 individuals (496 men and 501 women) with an average age of 37.71 years (*SD* = 10.62, ranging from 20 to 60 years). On average, participants reported 14.55 years of formal education (*SD* = 2.76), with the following current employment situation: 71% working full-time, 15% working part-time, 4% going to school, 1% retired, 4% unemployed, and 5% other. Most participants identified as Jewish (99%), with 59% being married, 21% being single, 8% exclusively dating, 6% cohabiting, 1% engaged, and 5% divorced. Regarding their self-reported current economic status, 7% described themselves as “below average”, 39% described themselves as “average”, and 54% described themselves as “above average”. Finally, on average, participants reported that the average length of their current romantic relationships is 10.76 years (*SD* = 8.52).

We did not pre-register this study, but the data file is available on the Open Science Framework (OSF) at: https://osf.io/4mf9q/ (accessed on 10 September 2024). This study had three phases: pre-manipulation, manipulation, and post-manipulation.

#### Manipulation Phase

During the pre-manipulation phase, participants were asked to complete questionnaires assessing their narcissistic and borderline personality traits. For the manipulation phase, participants were asked to:

*“Please think of a serious committed romantic relationship that you currently have, have had in the past, or would like to have in the future. Now that you are thinking about this committed romantic relationship, imagine the following scenario happening…”*

Participants were then randomly assigned to read a hypothetical scenario taken from Besser and Priel (2009) [13] and Cater et al. (2016) [16] that was intended to invoke either high or low levels of threat in their romantic relationships. Both scenarios began by describing the following situation:

*“You get out of work early and decide to surprise your partner and buy her/him a present. As you walk up to the apartment, you hear some laughing coming from inside. As you get closer, you see that the door is cracked open.”*

The scenarios diverge at this point with the high-threat scenario concluding with the following description:

*“You open the door to find your partner and another person having sexual relations in the living room. You hear your partner whispering to this person, ‘I think I might be in love with you.’”*

In contrast, the low-threat scenario concludes with this description:

*“You open the door to find your partner setting the table while the TV in the living room shows a couple laughing while they have sexual relations.”*

After reading the appropriate scenarios, participants were asked to complete measurements that would indicate their anticipated reactions to the events depicted in these scenarios. These measures assessed negative emotional responses and evaluations of the relationship.

### 2.2. Questionnaires

#### 2.2.1. Pre-Manipulation Measure

##### Narcissistic Personality Traits

We employed the short form of the *Five-Factor Narcissism Inventory* [40] to assess extraverted narcissism (16 items; “I like being noticed by others” [α = 0.78]), antagonistic narcissism (32 items; “I hate being criticized so much that I can’t control my temper when it happens” [α = 0.87]), and neurotic narcissism (12 items; “When people criticize me, I get embarrassed” [α = 0.79]). Responses were provided using scales that ranged from “*strongly disagree*” (1) to “*strongly agree*” (5).

##### Communal Narcissism

We used the *Communal Narcissism Inventory* [22] to capture communal narcissism (16 items, e.g., “I am the most helpful person I know” [α = 0.89]). Responses were provided using scales that ranged from “*disagree strongly*” (1) to “*agree strongly*” (5).

##### Borderline Personality Traits

We assessed borderline personality traits using the *Five Factor Borderline Inventory-Super Short Form* (FFBI-SSF) [28]. The FFBI-SSF (23 items; e.g., “I can be so different with different people that I wonder who I am” [α = 0.92]) is a shortened version of the original FFBI [41], which measures borderline personality traits from the framework of the Five Factor Model of personality. We decided to focus on the overall composite score for the FFBI-SSF.

#### 2.2.2. Post-Manipulation Measures

##### Negative Emotional Response

The anticipated negative emotional reaction of participants was assessed by asking them to report how they would anticipate feeling if they experienced the events described in the scenario (9 items; e.g., “I would be angry” [α = 0.95]). Participants rated their level of agreement with each item using scales that ranged from 1 (*disagree strongly*) to 5 (*agree strongly*).

##### Evaluations of the Relationship

We used the Investment Model Scale [42] to assess evaluations of the romantic relationship. Participants were asked to complete this measure based on how they believed they would feel about their relationships if they experienced the event described in the scenario. This instrument captures four aspects of romantic relationship functioning: satisfaction (5 items; e.g., *“Our relationship makes me very happy”* [α = 0.97]), investment in relationship (5 items; e.g., *“I have put a great deal into our relationship that I would lose if the relationship were to end”* [α = 0.86]), quality of alternatives (5 items; e.g., “*The people other than my partner with whom I might become involved are very appealing”* [α = 0.81]), and commitment (7 items; e.g., *“I want our relationship to last for a very long time”* [α = 0.92]). We chose to employ a comprehensive evaluation of the romantic relationship by calculating the average score of all questionnaire items, with items related to the quality of alternatives being reverse-scored (α = 0.93). Responses were made on scales ranging from 1 (*do not agree at all*) to 8 (*agree completely*).

### 2.3. Ethics Statement

Participation in this study was voluntary, and participants were aware that they could withdraw from the study at any time. All participants provided their signed, informed consent. No social security numbers or other identifying data were collected, nor were any invasive examinations conducted. This project was conducted with the approval of the Ethics Committee (IRB) of Hadassah Academic College.

### 2.4. Statistical Analysis

We began our analyses by examining the Pearson product-moment correlation coefficients among the variables. This was followed by a series of conditional process analyses (i.e., moderated mediation analyses) because we expected the threat condition (moderator) to moderate the associations that the personality traits (predictors) had with negative emotional reaction (mediator), which, in turn, would be associated with the evaluation of the romantic relationship (outcome). Because of the overlap between the personality traits, we were concerned that including all of them in the same analysis would make it difficult to understand how they were associated with the mediator and the outcome (see Lynam et al., 2006, for an extended discussion of this “perils of partialling” issue [43]). This prompted us to conduct separate conditional process analyses such that each personality trait served as the predictor in its own model. We used model 8 of the PROCESS macro [44] in conjunction with SPSS version 29 [45] to conduct these conditional process analyses. All of the variables were standardized to enhance the interpretability of the coefficients. We verified that the basic assumptions for these analyses were met prior to conducting these analyses (e.g., normally distributed residuals, homoscedasticity of the residuals, the absence of multicollinearity). For example, the Variance Inflation Factor (VIF) values were less than 1.63, which suggests that multicollinearity was not a concern. We conducted preliminary analyses that included gender as a moderator for exploratory purposes. However, gender did not emerge as a moderator in any of these analyses. As a result, we did not include gender in the final analyses, nor do we discuss gender differences in the interest of parsimony. It is important to note that although we used conditional process analyses to conceptualize the associations between these variables, we do not intend to infer causality because we are dealing with cross-sectional data that is correlational in nature.

## 3. Results

The correlation coefficients and descriptive statistics can be found in Table 1. Each personality trait had a small-to-medium positive correlation with negative emotional reaction in the low-threat condition, whereas neurotic narcissism and borderline personality traits were the only personality traits to be positively associated with negative emotional reaction in the high-threat condition. Antagonistic narcissism and borderline personality traits had medium negative correlations with the evaluation of the romantic relationship in the low-threat condition, whereas none of the personality traits were correlated with the evaluation of the romantic relationship in the high-threat condition.

As shown in Table 2, participants in the low-threat condition did not differ from those in the high-threat condition in terms of the personality traits. These results suggest that the random assignment of participants to the low-threat and high-threat conditions led to the groups having similar levels of these traits. As expected, participants in the high-threat condition reported higher levels of negative emotional reactions and lower evaluations of the romantic relationship than those in the low-threat condition.

### 3.1. Extraverted Narcissism

**Figure 1:**
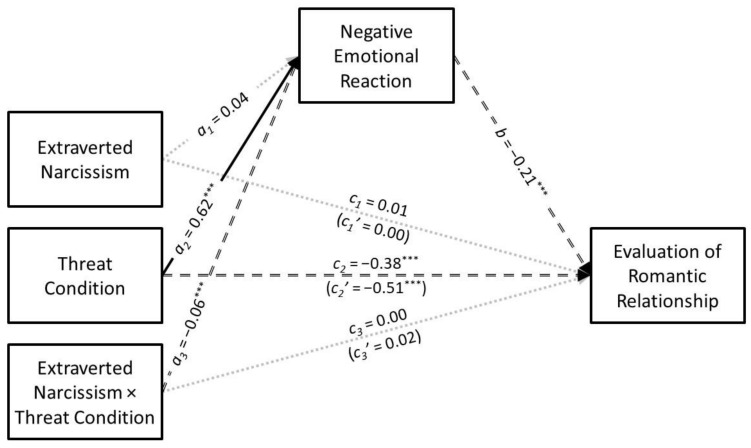
The results of the conditional process analysis showing whether threat condition moderated the indirect association that extraverted narcissism had with the evaluation of the romantic relationship through negative emotional reactions. Note: Solid black arrows represent significant positive associations. Dashed black arrows represent significant negative associations. Dotted grey arrows represent nonsignificant associations; *** *p* < 0.001.

**Figure 2:**
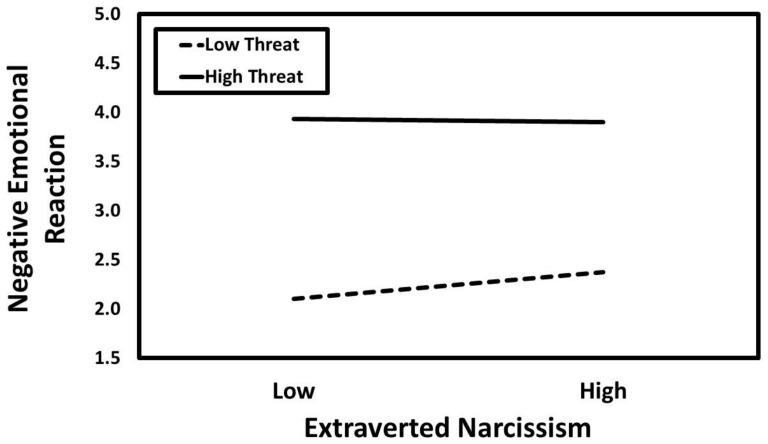
Predicted values illustrating the interaction that extraverted narcissism had with infidelity threat condition for negative emotional reaction. The predicted values for extraverted narcissism were estimated at values one standard deviation above and below its mean for high and low levels of extraverted narcissism, respectively.

The results of the conditional process analysis in which extraverted narcissism served as the predictor are presented in Figure 1. Extraverted narcissism was not associated with negative emotional reaction (*a*_1_ = 0.04, *SE* = 0.02, *t* = 1.75, *p* = 0.081, *CI*_95%_[−0.01, 0.09]), but its interaction with infidelity threat condition was significant (*a*_3_ = −0.06, *SE* = 0.02, *t* = −2.26, *p* = 0.024, *CI*_95%_[−0.11, −0.01]). The predicted values for the extraverted narcissism × infidelity threat interaction are presented in Figure 2. These predicted values are commonly used to visualize interaction effects because they represent the expected value of the outcome variable at specific levels of the predictor variables, based on the regression equation for the entire sample. When depicting interaction effects, it is common to present a continuous variable with “low” and “high” values—typically one standard deviation below and above the mean. This simplifies the interpretation of the interaction by showing how the association between the predictor variable and the outcome variable changes at distinct, interpretable levels of the moderator variable. Simple slopes tests revealed that extraverted narcissism had a positive association with negative emotional reaction in the low-threat condition (*B* = 0.24, *SE* = 0.09, *t* = 2.79, *p* = 0.005, *CI*_95%_[0.07, 0.41]) but not in the high-threat condition (*B* = −0.03, *SE* = 0.08, *t* = −0.38, *p* = 0.704, *CI*_95%_[−0.20, 0.13]).

Extraverted narcissism did not have a direct association with the evaluation of the romantic relationship (*c*_1_ = 0.01, *SE* = 0.03, *t* = 0.49, *p* = 0.621, *CI*_95%_[−0.04, 0.07]) nor was its interaction with infidelity threat condition significant (*c*_3_ = 0.00, *SE* = 0.03, *t* = 0.17, *p* = 0.869, *CI*_95%_[−0.05, 0.06]). Extraverted narcissism did not have an indirect association with the evaluation of the romantic relationship through negative emotional reaction (*a*_1_*b* = −0.01, *SE* = 0.01, *z* = −1.66, *p* = 0.097, *CI*_95%_[−0.02, 0.00]) nor did infidelity threat condition moderate this association (*B* = 0.02, *SE* = 0.01, *CI*_95%_[0.00, 0.05]).

### 3.2. Antagonistic Narcissism

**Figure 3:**
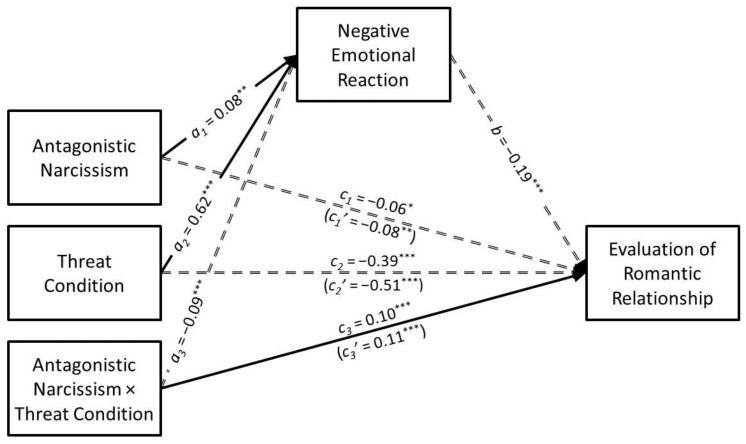
The results of the conditional process analysis showing whether threat condition moderated the indirect association that antagonistic narcissism had with the evaluation of the romantic relationship through negative emotional reactions. *Note*: Solid black arrows represent significant positive associations. Dashed black arrows represent significant negative associations. Dotted grey arrows represent nonsignificant associations. * *p* < 0.05; ** *p* < 0.01; *** *p* < 0.001.

**Figure 4:**
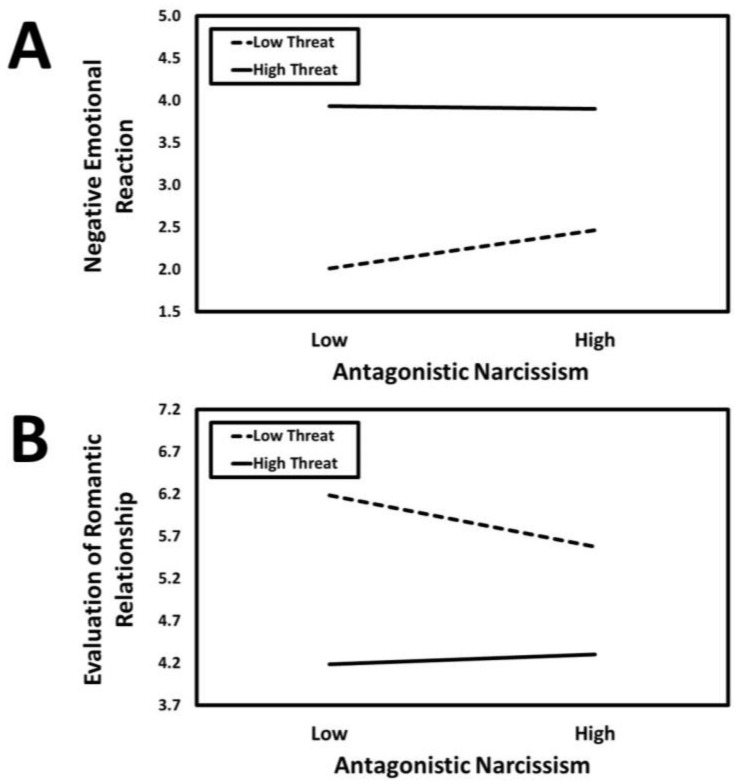
Predicted values illustrating the interaction that antagonistic narcissism had with infidelity threat condition for negative emotional reaction (**Panel A**) and evaluation of the romantic relationship (**Panel B**). The predicted values for antagonistic narcissism were estimated at values one standard deviation above and below its mean for high and low levels of antagonistic narcissism, respectively.

The results of the conditional process analysis in which antagonistic narcissism served as the predictor are presented in Figure 3. Antagonistic narcissism had a positive association with negative emotional reaction (*a*_1_ = 0.08, *SE* = 0.02, *t* = 3.13, *p* = 0.002, *CI*_95%_[0.03, 0.13]) that was moderated by infidelity threat condition (*a*_3_ = −0.09, *SE* = 0.02, *t* = −3.58, *p* < 0.001, *CI*_95%_[−0.14, −0.04]). The predicted values for the antagonistic narcissism × infidelity threat interaction are presented in Figure 4A. Simple slopes tests revealed that antagonistic narcissism had a positive association with negative emotional reaction in the low-threat condition (*B* = 0.46, *SE* = 0.18, *t* = 4.67, *p* < 0.001, *CI*_95%_[0.27, 0.65]) but not in the high-threat condition (*B* = −0.03, *SE* = 0.10, *t* = −0.34, *p* = 0.733, *CI*_95%_[−0.22, 0.15]).

Antagonistic narcissism had a negative direct association with the evaluation of the romantic relationship (*c*_1_ = −0.06, *SE* = 0.03, *t* = −2.28, *p* = 0.023, *CI*_95%_[−0.11, −0.01]) that was moderated by infidelity threat condition (*c*_3_ = 0.10, *SE* = 0.03, *t* = 3.60, *p* < 0.001, *CI*_95%_[0.04, 0.15]). The predicted values for the antagonistic narcissism × infidelity threat interaction are presented in Figure 4B. Simple slopes tests revealed that antagonistic narcissism was negatively associated with evaluation of the romantic relationship in the low-threat condition (*B* = −0.62, *SE* = 0.13, *t* = −4.86, *p* < 0.001, *CI*_95%_[−0.87, −0.37]) but not in the high-threat condition (*B* = 0.13, *SE* = 0.12, *t* = 1.02, *p* = 0.307, *CI*_95%_[−0.12, 0.37]).

Antagonistic narcissism had a negative indirect association with the evaluation of the romantic relationship through negative emotional reaction (*a*_1_*b* = −0.02, *SE* = 0.01, *z* = −2.69, *p* = 0.007, *CI*_95%_[−0.03, −0.01]). However, the indirect association was moderated by infidelity threat condition (*B* = 0.03, *SE* = 0.01, *CI*_95%_[0.01, 0.06]). More specifically, negative emotional reaction mediated the association that antagonistic narcissism had with the evaluation of the romantic relationship in the low-threat condition (*B* = −0.03, *SE* = 0.01, *CI*_95%_[−0.05, −0.01]) but not in the high-threat condition (*B* = 0.00, *SE* = 0.01, *CI*_95%_[−0.01, 0.01]).

### 3.3. Neurotic Narcissism

**Figure 5:**
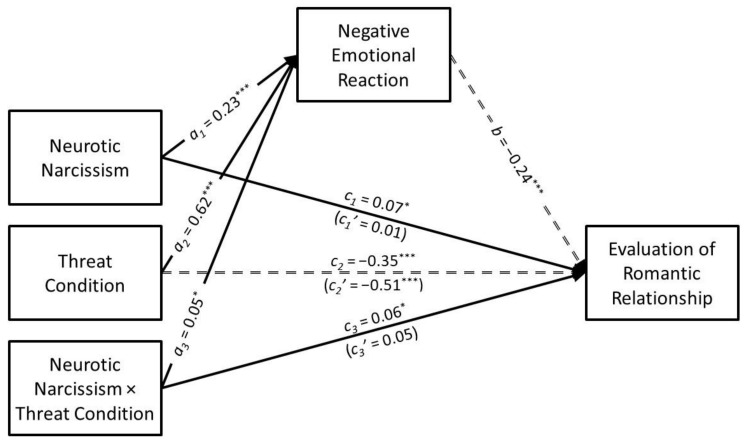
The results of the conditional process analysis showing whether threat condition moderated the indirect association that neurotic narcissism had with the evaluation of the romantic relationship through negative emotional reactions. *Note*: Solid black arrows represent significant positive associations. Dashed black arrows represent significant negative associations. Dotted grey arrows represent nonsignificant associations. * *p* < 0.05; *** *p* < 0.001.

**Figure 6:**
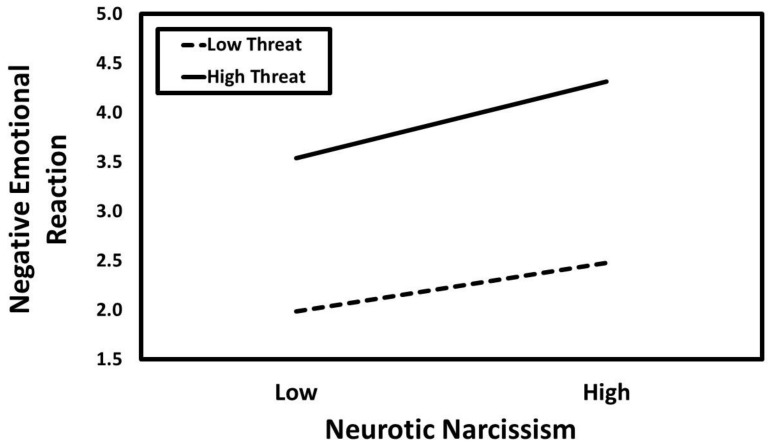
Predicted values illustrating the interaction that neurotic narcissism had with infidelity threat condition for negative emotional reaction. The predicted values for neurotic narcissism were estimated at values one standard deviation above and below its mean for high and low levels of neurotic narcissism, respectively.

The results of the conditional process analysis in which neurotic narcissism served as the predictor are presented in Figure 5. Neurotic narcissism had a positive association with negative emotional reaction (*a*_1_ = 0.23, *SE* = 0.02, *t* = 9.75, *p* < 0.001, *CI*_95%_[0.19, 0.28]) that was moderated by infidelity threat condition (*a*_3_ = 0.05, *SE* = 0.02, *t* = 2.18, *p* = 0.030, *CI*_95%_[0.01, 0.10]). The predicted values for the neurotic narcissism × infidelity threat interaction are presented in Figure 6. Simple slopes tests revealed that neurotic narcissism had a positive association with negative emotional reaction in the low-threat condition (*B* = 0.38, *SE* = 0.07, *t* = 5.38, *p* < 0.001, *CI*_95%_[0.24, 0.52]) but this association was especially strong in the high-threat condition (*B* = 0.61, *SE* = 0.07, *t* = 8.37, *p* < 0.001, *CI*_95%_[0.46, 0.75]).

Neurotic narcissism had a positive direct association with the evaluation of the romantic relationship (*c*_1_ = 0.07, *SE* = 0.03, *t* = 2.51, *p* = 0.012, *CI*_95%_[0.02, 0.13]) but this association was not moderated by infidelity threat condition (*c*_3_ = 0.05, *SE* = 0.03, *t* = 1.91, *p* = 0.057, *CI*_95%_[0.00, 0.10]). Neurotic narcissism had a negative indirect association with the evaluation of the romantic relationship through negative emotional reaction (*a*_1_*b* = −0.06, *SE* = 0.01, *z* = −5.56, *p* < 0.001, *CI*_95%_[−0.08, −0.04]). However, the indirect association was moderated by infidelity threat condition (*B* = −0.03, *SE* = 0.01, *CI*_95%_[−0.05, −0.01]). More specifically, negative emotional reaction mediated the association that neurotic narcissism had with the evaluation of the romantic relationship in the low-threat condition (*B* = −0.04, *SE* = 0.01, *CI*_95%_[−0.07, −0.02]) but this indirect association was even stronger in the high-threat condition (*B* = −0.07, *SE* = 0.01, *CI*_95%_[−0.09, −0.05]).

### 3.4. Communal Narcissism

**Figure 7:**
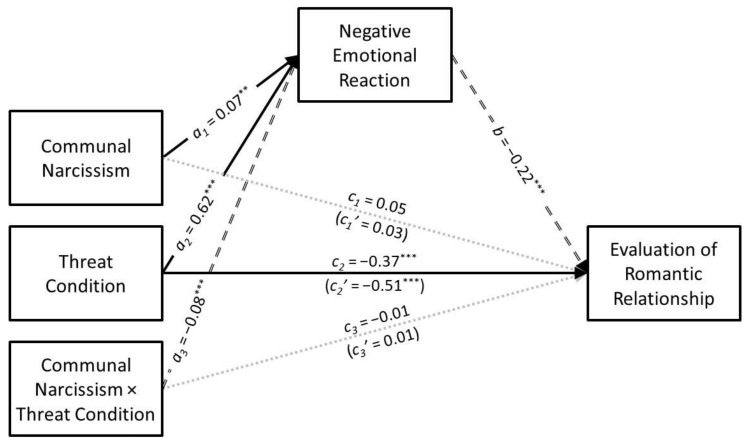
The results of the conditional process analysis showing whether threat condition moderated the indirect association that communal narcissism had with the evaluation of the romantic relationship through negative emotional reactions. *Note*: Solid black arrows represent significant positive associations. Dashed black arrows represent significant negative associations. Dotted grey arrows represent nonsignificant associations. ** *p* < 0.01; *** *p* < 0.001.

**Figure 8:**
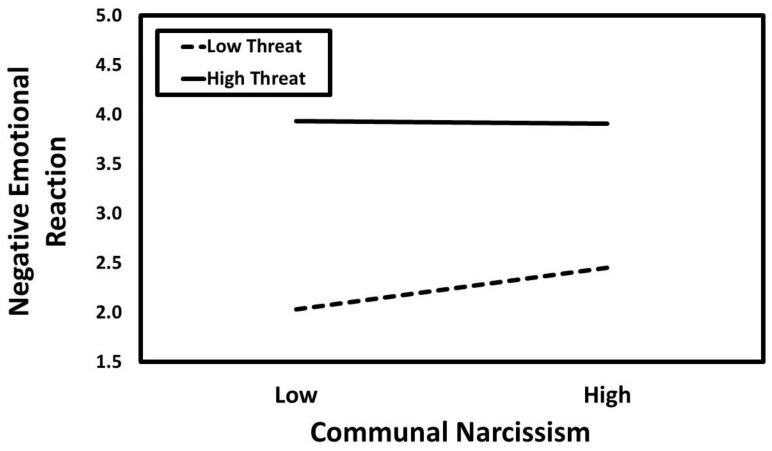
Predicted values illustrating the interaction that communal narcissism had with infidelity threat condition for negative emotional reaction. The predicted values for communal narcissism were estimated at values one standard deviation above and below its mean for high and low levels of communal narcissism, respectively.

The results of the conditional process analysis in which communal narcissism served as the predictor are presented in Figure 7. Communal narcissism had a positive association with negative emotional reaction (*a*_1_ = 0.07, *SE* = 0.02, *t* = 2.93, *p* = 0.003, *CI*_95%_[0.02, 0.12]) that was moderated by infidelity threat condition (*a*_3_ = −0.08, *SE* = 0.02, *t* = −3.34, *p* < 0.001, *CI*_95%_[−0.13, −0.03]). The predicted values for the communal narcissism × infidelity threat interaction are presented in Figure 8. Simple slopes tests revealed that communal narcissism had a positive association with negative emotional reaction in the low-threat condition (*B* = 0.22, *SE* = 0.05, *t* = 4.39, *p* < 0.001, *CI*_95%_[0.12, 0.32]) but not in the high-threat condition (*B* = −0.02, *SE* = 0.05, *t* = −0.31, *p* = 0.758, *CI*_95%_[−0.11, 0.08]).

Communal narcissism did not have a direct association with the evaluation of the romantic relationship (*c*_1_ = 0.05, *SE* = 0.03, *t* = 1.75, *p* = 0.080, *CI*_95%_[−0.01, 0.10]) nor was its interaction with infidelity threat condition significant (*c*_3_ = −0.01, *SE* = 0.03, *t* = −0.21, *p* = 0.838, *CI*_95%_[−0.06, 0.05]). Communal narcissism had a negative indirect association with the evaluation of the romantic relationship through negative emotional reaction (*a*_1_*b* = −0.02, *SE* = 0.01, *z* = −2.63, *p* = 0.009, *CI*_95%_[−0.03, −0.01]). However, the indirect association was moderated by infidelity threat condition (*B* = 0.04, *SE* = 0.01, *CI*_95%_[0.01, 0.06]). More specifically, negative emotional reaction mediated the association that communal narcissism had with the evaluation of the romantic relationship in the low-threat condition (*B* = −0.03, *SE* = 0.01, *CI*_95%_[−0.06, −0.01]) but not in the high-threat condition (*B* = 0.00, *SE* = 0.01, *CI*_95%_[−0.01, 0.02]).

### 3.5. Borderline Personality Traits

**Figure 9:**
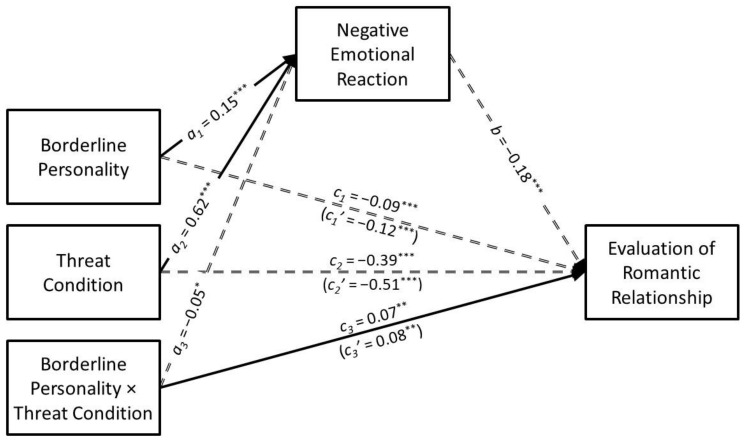
The results of the conditional process analysis showing whether threat condition moderated the indirect association that borderline personality traits had with the evaluation of the romantic relationship through negative emotional reactions. *Note*: Solid black arrows represent significant positive associations. Dashed black arrows represent significant negative associations. Dotted grey arrows represent nonsignificant associations. * *p* < 0.05; ** *p* < 0.01; *** *p* < 0.001.

**Figure 10:**
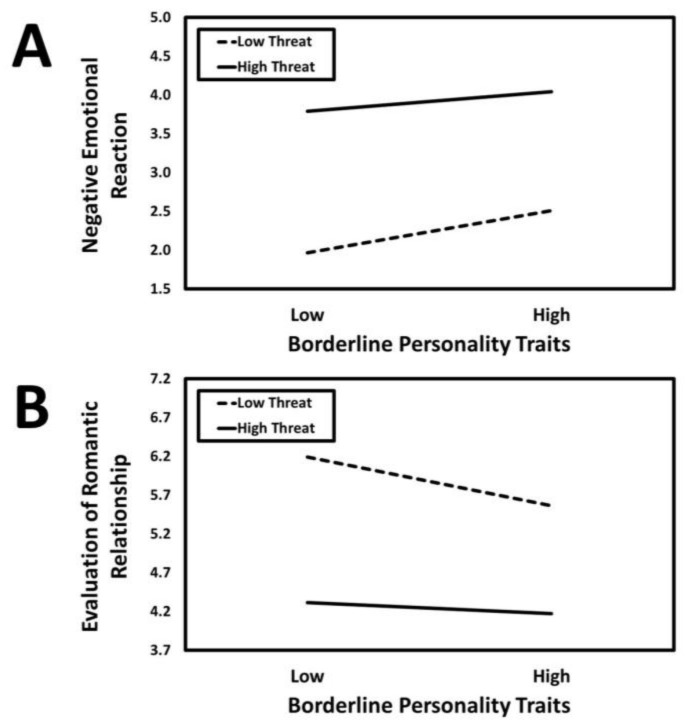
Predicted values illustrating the interaction that borderline personality traits had with infidelity threat condition for negative emotional reaction (**Panel A**) and evaluation of the romantic relationship (**Panel B**). The predicted values for borderline personality traits were estimated at values one standard deviation above and below its mean for high and low levels of borderline personality traits, respectively.

The results of the conditional process analysis in which borderline personality traits served as the predictor are presented in Figure 9. Borderline personality traits had a positive association with negative emotional reaction (*a*_1_ = 0.15, *SE* = 0.02, *t* = 6.00, *p* < 0.001, *CI*_95%_[0.10, 0.19]) that was moderated by infidelity threat condition (*a*_3_ = −0.05, *SE* = 0.02, *t* = −2.18, *p* = 0.030, *CI*_95%_[−0.10, −0.01]). The predicted values for the borderline personality traits × infidelity threat interaction are presented in Figure 10A. Simple slopes tests revealed that borderline personality traits had a positive association with negative emotional reaction in the high-threat condition (*B* = 0.19, *SE* = 0.07, *t* = 2.69, *p* = 0.007, *CI*_95%_[0.05, 0.33]) but this association was especially strong in the low-threat condition (*B* = 0.41, *SE* = 0.07, *t* = 5.76, *p* < 0.001, *CI*_95%_[0.27, 0.55]).

Borderline personality traits had a negative direct association with the evaluation of the romantic relationship (*c*_1_ = −0.09, *SE* = 0.03, *t* = −3.38, *p* < 0.001, *CI*_95%_[−0.14, −0.04]) that was moderated by infidelity threat condition (*c*_3_ = 0.07, *SE* = 0.03, *t* = 2.61, *p* = 0.009, *CI*_95%_[0.02, 0.12]). The predicted values for the borderline personality traits × infidelity threat interaction are presented in Figure 10B. Simple slopes tests revealed that borderline personality traits were negatively associated with evaluation of the romantic relationship in the low-threat condition (*B* = −0.48, *SE* = 0.09, *t* = −5.17, *p* < 0.001, *CI*_95%_[−0.67, −0.30]) but not in the high-threat condition (*B* = −0.09, *SE* = 0.09, *t* = −1.01, *p* = 0.313, *CI*_95%_[−0.28, 0.09]).

Borderline personality traits had a negative indirect association with the evaluation of the romantic relationship through negative emotional reaction (*a*_1_*b* = −0.03, *SE* = 0.01, *z* = −3.94, *p* < 0.001, *CI*_95%_[−0.04, −0.01]). However, this indirect association was not moderated by infidelity threat condition (*B* = 0.02, *SE* = 0.01, *CI*_95%_[0.00, 0.04]).

## 4. Discussion

This study explored the connections that narcissistic and borderline personality traits had with reactions to potential infidelity, providing valuable insights into how these traits affect emotional and evaluative responses in romantic contexts. Our results revealed that neurotic narcissism was significantly associated with heightened negative emotional responses across both scenarios, particularly under high-threat conditions. This finding is consistent with previous research that highlights neurotic narcissism’s inherent emotional distress and need for validation, making individuals highly reactive to perceived betrayal [5,14]. Given these intense emotional reactions, it is unsurprising that neurotic narcissism was indirectly linked to more negative evaluations of the romantic relationship. Individuals with neurotic narcissism may view their partner’s imagined sexual infidelity as a direct threat to their self-worth, amplifying emotional volatility and decreasing their overall relationship satisfaction. This heightened sensitivity to perceived betrayal likely undermines their ability to maintain a positive view of the relationship in such contexts. Consequently, neurotic narcissism’s negative emotional impact appears to drive more pessimistic assessments of romantic relationships in the face of perceived infidelity.

In contrast to what was observed for neurotic narcissism, antagonistic narcissism—which is characterized by defensiveness and hostility—was notably associated with negative evaluations of romantic relationships in the low-threat condition but not in the high-threat condition. This suggests that individuals with antagonistic narcissism may perceive minor relational threats as significant, exacerbating relationship dissatisfaction [20]. One potential explanation for this pattern is that personality traits, such as antagonistic narcissism, often exert a stronger influence in relatively “weak” situations, where there is little external pressure to respond in a particular way [44]. In such conditions, individuals’ personality traits may guide their emotional responses, leading to divergent reactions. In contrast, high-threat situations, like imagining sexual infidelity, may act as “strong” situations that elicit similarly negative emotional reactions across individuals, regardless of personality traits. In these highly stressful scenarios, individuals are likely to experience intense emotional distress regardless of their level of antagonistic narcissism, minimizing the connections that personality traits have with outcomes such as relationship evaluations. The findings suggest that antagonistic narcissism’s detrimental effects on emotional reactions and relationship functioning are more likely to emerge when the relational threat is perceived as ambiguous or minor, allowing for greater variability in responses influenced by personality.

Extraverted narcissism, defined by self-assurance and the need for admiration, did not show significant associations with emotional reactions or relationship evaluations in either threat condition. This indicates that the external focus of extraverted narcissism may mitigate internal emotional impacts when facing potential relational threats. These findings are in line with a previous study conducted by Besser and Priel (2010) which found that grandiose narcissism (which is similar to extraverted narcissism) is associated with heightened reactivity to achievement setbacks, whereas vulnerable narcissism (which is similar to neurotic narcissism) involves sensitivity to shaming interpersonal experiences [14].

Communal narcissism, which involves projecting a façade of altruism and helpfulness, was associated with negative emotional responses primarily in low-threat conditions. This indicates that despite appearing supportive, communal narcissists may experience some level of internal distress during periods of relative calm in the relationship [22]. They may also feel conflicted when their self-perceived altruism is questioned, leading to resentment or frustration towards their partners, thereby affecting the quality of the relationship.

Borderline personality traits were linked to strong emotional reactivity across both threat conditions, with responses particularly pronounced in low-threat scenarios. This aligns with existing literature on the emotional instability and abandonment fears characteristic of borderline traits. One potential explanation for this finding is that individuals with borderline personality traits may experience even minor relational ambiguities as significant emotional threats, leading to heightened negative emotional responses and more negative evaluations of their romantic relationship. In low-threat situations, where the pressure to respond in a specific way is reduced, these individuals may project their internal fears of abandonment onto relatively neutral or ambiguous circumstances, magnifying their emotional reactions [24]. In contrast, high-threat scenarios, such as imagined infidelity, may trigger strong negative responses across individuals regardless of their personality traits, reducing the relative impact of borderline personality traits in these situations. The findings suggest that individuals with borderline traits are particularly vulnerable to emotional dysregulation in less overtly threatening relational contexts, which can significantly erode their relationship satisfaction and overall evaluations of the relationship.

An intriguing aspect of our findings relates to interpreting the potential stress associated with low-threat conditions. It seems that the low-threat vs. high-threat scenarios could be differentiated in terms of being perceived as a state of uncertainty (What if there is another person in his/her life?—suspicion-related long-term distress) versus a state of certainty (there is another person in his/her life, and they are having sex—“reality shock”, traumatic overwhelming short-term distress). Living with the suspicion that one’s partner is being unfaithful may be nearly as upsetting as experiencing confirmed betrayal. Our study suggests the possibility that even minor suggestions of potential infidelity—such as those included in our low-threat condition—may be upsetting for individuals with certain personality traits. This highlights how individuals with certain personality traits may interpret ambiguous situations in a maladaptive manner that triggers various social-cognitive processes (e.g., defensiveness, feeling unappreciated, withdrawing from the relationship, engaging in passive-aggressive behaviors) that may be detrimental to their relationship functioning. However, it seems that infidelity is such a potent threat that it overwhelms the impact of the examined personality traits (except for neurotic narcissism).

This aligns with the observation that personality traits often have stronger associations with outcomes in situations that are relatively “weak” (i.e., involve little pressure to respond in a particular way) compared to those that are “strong” (i.e., exert considerable pressure to respond in a particular way) [46]. That is, weak situations (such as our low-threat condition) may allow for divergent responses which may be guided in part by personality traits, whereas strong situations (such as our high-threat condition) may lead to similar responses across individuals regardless of their personality traits. In essence, the infidelity of one’s romantic partner may be such a strong situation that it elicits negative reactions regardless of certain personality traits. These findings show that the connections that some of the personality traits had with relationship functioning could only be observed under “low-threat” conditions (e.g., antagonistic narcissism). Many of these personality traits did not seem to be particularly detrimental in terms of emotional reactions or evaluations of the relationship in the high-threat situations, as everyone was so upset by the infidelity scenario, since a real or imagined extradyadic rival poses an outside threat to an established relationship and is known to be a highly stressful and traumatic event (e.g., [47]).

Previous research demonstrates that the mere suspicion of a partner’s infidelity can lead to powerful psychological, physical, and behavioral consequences within romantic relationships [48]. Evidence suggests that suspecting a partner of cheating is linked to harmful outcomes for the individual, such as increased frequency and severity of health problems [49], depression [50], and higher engagement in risky behaviors, including alcohol and drug use [51]. These findings imply that the belief in potential betrayal can elicit levels of distress similar to those experienced after an actual betrayal and according to our study, especially among individuals high on borderline personality features as well as several narcissistic personality features such as antagonistic and communal narcissism.

When individuals suspect their partners of infidelity, they often confront uncertainty about the status and future of their relationship, leading to a growing distrust of their partner (e.g., [52]). This uncertainty can intensify negative emotions [53] and may escalate relationship conflicts, foster aggression, provoke retaliatory behaviors, and even contribute to relationship dissolution [54]. Consequently, the suspicion of a partner’s unfaithfulness creates a highly stressful environment. Individuals who possess elevated levels of borderline personality traits are known to be characterized by suspicious jealousy [39].

Transactional stress theory [55] provides a valuable framework for understanding the stress associated with these low-threat suspicions. According to this theory, stress arises when individuals perceive that the demands of a situation exceed their ability to cope. The effect of a stressor is influenced by the individual’s appraisal of the situation and various personal and environmental factors that can affect the intensity of the stress experienced. A critical point of the theory is that the way an individual appraises a situation shapes the stress consequences linked to it. For suspicions of a partner’s infidelity to become harmful, they must first be appraised as distressing. Additionally, anticipatory appraisals—concerns about what may happen—can also generate distress [55,56,57]. Thus, even the mere possibility of infidelity can induce stress, leading to heightened feelings of distress when specific individuals perceive such suspicion as a threat.

Multiple lines of research support the notion that even suspicions of infidelity can evoke stress. First, whether real or imagined, the presence of an extradyadic rival poses a threat to an established relationship, indicating potential loss and creating a highly stressful experience [47,58,59]. Second, losing a romantic partner to someone else often feels like rejection, diminishing self-esteem and leading individuals to feel unlovable or insecure in their ability to retain their partner’s affection (e.g., [60]). Third, the possibility of infidelity typically violates established norms within most romantic relationships. The majority of individuals view infidelity as unacceptable in monogamous contexts [61], and when such norms are breached, it can evoke feelings of hurt and personal injury [52]. Additionally, individuals possess mental models of healthy romantic relationships, and the prospect of betrayal can challenge these expectations [52]. Finally, a partner’s potential infidelity may imply a devaluation of the relationship, suggesting a lower level of commitment [62].

Based on these findings, our study proposes that higher levels of suspicion regarding a partner’s betrayal, especially among individuals with elevated levels of borderline personality features or antagonistic narcissism, are likely correlated with increased perceived distress. This understanding highlights the serious implications of suspected infidelity, even in low-threat contexts, for individual well-being and relationship stability.

Our findings regarding the impact of a high level of infidelity threat as traumatic for certain personality traits, are in line with research which shows that high betrayal trauma significantly impacts personality pathology, particularly beyond other trauma types, with a focus mainly on borderline personality disorder, and recent studies suggest it may also affect other personality disorders, like pathological narcissism (see [63] for review). Our results also extend prior research by elucidating the differential effects of narcissistic traits in varying threat contexts. While previous studies have established a link between narcissism and infidelity [6,9], our study demonstrates that perceived levels of infidelity threat from one’s partner influence the connections these personality traits have with emotional and relational outcomes.

These findings have important clinical implications. Tailoring interventions to the specific narcissistic tendencies can improve relationship dynamics. For example, enhancing emotional resilience in neurotic narcissists may be crucial in crisis situations, whereas addressing defensive behaviors in antagonistic individuals may be beneficial under everyday low-threat conditions. Therapists might implement techniques such as cognitive-behavioral strategies to help neurotic narcissists reframe their thoughts and reduce sensitivity to perceived relational threats. For antagonistic narcissists, therapists could focus on fostering open communication skills and emotional awareness to mitigate defensive responses. For those with borderline personality traits, interventions focused on emotional regulation could alleviate pervasive distress. Therapists could introduce mindfulness techniques or dialectical behavior therapy (DBT) skills to help these individuals manage their emotional reactivity and reduce the fear of abandonment.

Limitations: The use of hypothetical scenarios in this study, while providing control, may not fully capture the intensity or complexity of real-life infidelity situations. Participants might react differently in a real situation due to the strength of the emotions and the complexities of the relational dynamics that are involved. Recognizing that real-life contexts involve a multitude of variables—such as ongoing relationship history, contextual factors, and individual life experiences—could significantly alter reactions to infidelity by one’s romantic partner. Addressing these limitations in future studies will help solidify the applicability of the findings to clinical practice. Specifically, longitudinal research exploring actual instances of infidelity could provide deeper insights into how personality traits are connected with relationship dynamics in real-time. It is also important to note that we focused on negative emotional responses to infidelity (e.g., anger, anxiety, depression), but we did not include specific references to feelings of jealousy in the present study. Future research should investigate the role that feelings of jealousy play in how individuals with narcissistic and borderline personality traits respond to infidelity by their partners. This may be a particularly beneficial course for future research because previous studies have shown that issues surrounding jealousy are important for understanding the connections that narcissistic personality traits [64] and borderline personality traits [39] have with various aspects of romantic relationship functioning.

The sample consisted predominantly of Israeli participants, with 99% identifying as Jewish. This cultural homogeneity may limit the generalizability of the findings to other cultural or religious contexts where views on infidelity and personality expression might differ. Additionally, the reliance on self-reported questionnaires introduces the possibility of social desirability bias and inaccurate self-assessment, particularly regarding sensitive topics such as personality traits and relationship dynamics. The study’s cross-sectional nature limits causal interpretations of the relationships between personality traits and reactions to infidelity. Longitudinal studies could provide deeper insights into how these dynamics evolve over time.

To broaden the understanding of how cultural factors influence the relationship between personality traits and responses to infidelity, future research should involve more diverse cultural samples. Investigating real-life instances of infidelity, rather than relying on hypothetical scenarios, is also crucial for gaining accurate and applicable insights because it captures the genuine emotional and behavioral responses of individuals. Additionally, further studies should explore how factors like relationship length, attachment styles, or commitment levels might moderate the impact of personality traits on reactions to infidelity. It would also be interesting to see how individuals who have engaged in infidelity themselves respond to this sort of potential infidelity from their partner. Research using milder forms of relational threats, such as emotional withdrawal or perceived flirtation, would help provide a more nuanced understanding about how personality traits are linked with responses in less severe but more common relational situations. Longitudinal designs are recommended to assess how personality traits influence the long-term dynamics of relationships, particularly following infidelity, to better understand causal pathways and changes over time. Exploring how these personality traits relate to a broader range of behavioral responses, such as communication styles, relationship repair efforts, or maladaptive behaviors like intimate partner violence, could expand our understanding of personality influences in relational contexts. Additionally, by studying trait dynamics with weaker relational threat manipulations [13], future research could clarify the interaction between personality traits and perceived threat levels and examine their connections to impulsive retaliatory behaviors or intimate partner violence, providing a comprehensive understanding of their relational impact.

## 5. Conclusions

Despite its limitations, this study advances the understanding of how narcissistic and borderline personality traits are associated with emotional and relational responses to perceived romantic betrayal. By highlighting the importance of threat context, specifically the suspected infidelity distress, the findings underscore the need for personalized therapeutic approaches to address the varied impacts of these traits in romantic relationships. The findings of the study carry significant implications for clinical practice, especially for practitioners working with patients exhibiting notable levels of narcissistic and borderline personality traits. It suggests that trauma marked by high levels of betrayal may affect both the vulnerabilities these patients defend against and the grandiose defenses they utilize. Acknowledging the role of trauma in the defensive behaviors of narcissistic individuals can inform the incorporation of trauma-focused interventions alongside methods aimed at modifying personality organization. Furthermore, this study emphasizes the need for careful consideration in couple therapy for participants with vulnerable, grandiose, and communal narcissism. Specifically, the distress related to suspected infidelity should be addressed with sensitivity, as it may have serious implications for both mental health and relationship stability. Future research should investigate how to effectively combine personality-focused approaches with trauma-specific strategies in psychotherapy.

## Figures and Tables

**Table 1:** Intercorrelations and Descriptive Statistics.

	1	2	3	4	5	6	7
1. Extraverted Narcissism	–	0.39 ***	−0.15 **	0.55 ***	0.00	−0.02	0.02
2. Antagonistic Narcissim	0.39 ***	–	0.07	0.22 ***	0.51 ***	−0.02	0.04
3. Neurotic Narcissism	−0.13 **	0.02	–	−0.17 ***	0.35 ***	0.44 ***	0.07
4. Communal Narcissism	0.59 ***	0.32 ***	−0.11 *	–	−0.01	−0.02	0.05
5. Borderline Personality	0.02	0.46 ***	0.37 ***	0.04	–	0.15 **	−0.04
6. Negative Emotional Reaction	0.11 *	0.18 ***	0.20 ***	0.17 ***	0.22 ***	–	−0.03
7. Evaluation of Relationship	−0.02	−0.23 ***	−0.05	0.02	−0.24 ***	−0.33 ***	–
*Mean _Low-Threat Condition_*	3.26	2.47	3.21	3.88	1.89	2.24	5.88
*Standard Deviation _Low-Threat Condition_*	0.55	0.49	0.64	0.94	0.66	1.23	1.31
*Skewness _Low-Threat Condition_*	−0.43	0.19	−0.12	−0.04	0.70	0.72	−0.61
*Kurtosis _Low-Threat Condition_*	0.02	−0.25	−0.32	−0.19	−0.20	−0.69	−0.20
*Mean _High-Threat Condition_*	3.28	2.46	3.18	3.88	1.89	3.92	4.25
*Standard Deviation _High-Threat Condition_*	0.57	0.50	0.63	0.96	0.67	0.88	1.47
*Skewness _High-Threat Condition_*	−0.25	0.17	−0.01	−0.14	0.73	−0.80	0.47
*Kurtosis _High-Threat Condition_*	−0.12	−0.17	−0.32	0.05	−0.20	0.34	−0.65

*Note*. The values below the diagonal are taken from participants in the low-threat condition, whereas the values above the diagonal are taken from participants in the high-threat condition. * *p* < 0.05; ** *p* < 0.01; *** *p* < 0.001.

**Table 2:** Comparisons of the low-threat and high-threat conditions.

	Low-Threat Condition (*n* = 497)		High-Threat Condition (*n* = 500)		
	*M*	*SD*	*M*	*SD*	*t*
Extraverted Narcissism	3.26	0.55	3.28	0.57	0.42
Antagonistic Narcissism	2.47	0.49	2.46	0.50	−0.32
Neurotic Narcissism	3.21	0.64	3.18	0.63	−0.77
Communal Narcissism	3.88	0.94	3.88	0.98	0.03
Borderline Personality	1.89	0.66	1.89	0.67	0.02
Negative Emotional Reaction	2.24	1.23	3.92	0.88	24.78 *
Evaluation of Relationship	5.88	1.31	4.24	1.47	18.51 *

* *p* < 0.001.

## Data Availability

The data presented in this study are available on request from the corresponding authors.
